# Infrared Photography: A Novel Diagnostic Approach for Ocular Surface Abnormalities Due to Vitamin A Deficiency

**DOI:** 10.3390/diagnostics15151910

**Published:** 2025-07-30

**Authors:** Hideki Fukuoka, Chie Sotozono

**Affiliations:** Department of Ophthalmology, Kyoto Prefectural University of Medicine, Kyoto 602-8566, Japan; csotozon@koto.kpu-m.ac.jp

**Keywords:** vitamin A deficiency, infrared photography, conjunctival changes, pediatric nutrition, ocular surface, diagnostic imaging

## Abstract

Vitamin A deficiency (VAD) remains a significant cause of preventable blindness worldwide, with ocular surface changes representing early manifestations that require prompt recognition and treatment. Conventional examination methods are capable of detecting advanced changes; however, subtle conjunctival abnormalities may be overlooked, potentially delaying the administration of appropriate interventions. We herein present the case of a 5-year-old Japanese boy with severe VAD due to selective eating patterns. This case demonstrates the utility of infrared photography as a novel diagnostic approach for detecting and monitoring conjunctival surface abnormalities. The patient exhibited symptoms including corneal ulcers, night blindness, and reduced visual acuity. Furthermore, blood tests revealed undetectable levels of vitamin A (5 IU/dL), despite relatively normal physical growth parameters. Conventional slit-lamp examination revealed characteristic sandpaper-like conjunctival changes. However, infrared photography (700–900 nm wavelength) revealed distinct abnormal patterns of conjunctival surface folds and keratinization that were not fully appreciated on a routine examination. Following high-dose vitamin A supplementation (4000 IU/day), complete resolution of ocular abnormalities was achieved within 2 months, with infrared imaging objectively documenting treatment response and normalization of conjunctival surface patterns. This case underscores the potential for severe VAD in developed countries, particularly in the context of dietary restrictions, thereby underscoring the significance of a comprehensive dietary history and a meticulous ocular examination. Infrared photography provides a number of advantages, including the capacity for non-invasive assessment, enhanced visualization of subtle changes, objective monitoring of treatment response, and cost-effectiveness due to the use of readily available equipment. This technique represents an underutilized diagnostic modality with particular promise for screening programs and clinical monitoring of VAD-related ocular manifestations, potentially preventing irreversible visual loss through early detection and intervention.

**Figure 1:**
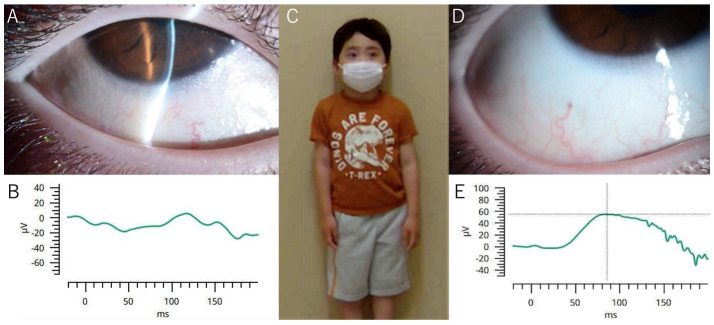
The following study will examine ocular manifestations and treatment response in a pediatric case of vitamin A deficiency (VAD). A slit-lamp photograph of the right eye was taken before treatment, and it exhibited an atypical, sandpaper-like appearance with bright reflections and keratinization on the conjunctival surface (**A**). A thorough evaluation of the electroretinogram (ERG) of the right eye was conducted prior to the initiation of treatment, which revealed severely attenuated scotopic responses, indicating a significant reduction in rod photoreceptor function. The amplitude of the b-wave is significantly reduced, which is consistent with the manifestation of VAD-related retinal dysfunction (**B**). A full-body photograph of the 5-year-old patient is presented, showing a relatively normal physical appearance despite the patient’s multiple nutritional deficiencies. His height (108 cm, low-normal) and weight (19.9 kg, high-normal) demonstrate the potential for severe VAD even in the context of apparently normal growth parameters (**C**). A slit-lamp photograph of the right eye was taken two months after the subject was given 4000 IU of vitamin A per day. The photograph shows that the conjunctival abnormalities have completely resolved. The conjunctival surface appears smooth, moist, and transparent without signs of keratinization or inflammation (**D**). A subsequent electroretinography (ERG) of the right eye was conducted after a two-month period of treatment using the RETeval system (LKC Technologies, Gaithersburg, MD, USA), which indicated a complete recovery of rod photoreceptor responses. The b-wave amplitude has been restored to its normal range, indicating the reestablishment of retinal function and the resolution of night blindness (**E**). A 5-year-old Japanese boy was admitted to the hospital with a urinary tract infection. During a subsequent ophthalmological examination, significant ocular abnormalities were detected. The patient exhibited corneal ulcers, edema, and abnormal conjunctival luster in both eyes (Figure 1A), with visual acuity reduced to 20/33 and 20/25 in the right and left eyes, respectively. He had recently developed night blindness with reduced rod response on electroretinography (ERG) (Figure 1B). A thorough review of the patient’s medical history revealed a diet marked by significant restrictions, with the patient adhering strictly to a diet consisting of white foods such as rice, bread, French fries, and noodles. This diet was further characterized by avoidance of all colored foods, indicating a markedly monochromatic dietary pattern. The results of the blood tests indicated undetectable levels of vitamin A of 5 IU/dL, in addition to deficiencies in vitamin D, selenium, iron, zinc, and carnitine. Despite these multiple nutritional deficiencies, physical examination showed relatively normal stature with height and weight of 108 cm (low-normal) and 19.9 kg (high-normal) (Figure 1C), respectively, indicating that physical appearance alone cannot exclude significant metabolic issues.

**Figure 2:**
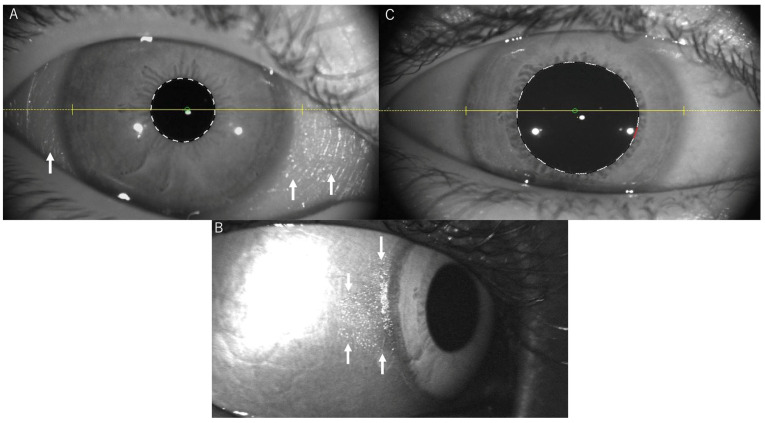
Infrared photography demonstrates conjunctival surface abnormalities in vitamin A deficiency and monitors treatment response. Pre-treatment images using the CASIA anterior segment OCT system reveal folds and abnormal conjunctival surfaces (white arrows) (**A**). After two weeks of vitamin A supplementation, Heidelberg Retina Tomograph 2 (HRT2) infrared imaging shows marked improvement of the reflective regions indicating conjunctival keratinization (white arrows) (**B**). Complete normalization is evident after 2 months of treatment using the CASIA anterior segment OCT system (**C**). Although infrared photography has been employed in various ophthalmological applications for decades [1,2], its specific utility in detecting subtle ocular surface abnormalities merits greater attention. Infrared photography, with a wavelength ranging from 700 to 900 nanometers, has been shown to provide enhanced visualization of subtle ocular surface changes that may not be fully apparent during routine examination. Pre-treatment infrared imaging revealed distinct abnormal patterns of conjunctival surface folds and keratinization, appearing as bright, reflective areas (Figure 2A, white arrows). These findings were more pronounced and clearly delineated in comparison with conventional photography. The infrared wavelength displays distinct penetration characteristics in comparison with visible light. This property enables the detection of structural abnormalities, including micro folding, early fibrosis, and areas of altered epithelial integrity. This detection occurs prior to the manifestation of these abnormalities with conventional techniques [3]. The difficulty in clearly visualizing conjunctival keratinization with conventional photography, even for experienced ophthalmologists, emphasizes the clinical value of infrared imaging as a more sensitive diagnostic modality for detecting subtle ocular surface changes in vitamin A deficiency. The therapeutic regimen was initiated with high-dose oral vitamin A supplementation at 4000 IU/day (200 IU/kg/day). While WHO guidelines recommend high-dose vitamin A treatment (200,000 IU on days 1, 2, and 14) for severe vitamin A deficiency with corneal involvement, our patient had already shown improvement with over-the-counter cod liver oil supplementation (1524 IU/day) initiated by the ophthalmology department. Subsequently, the patient was maintained on 4000 IU/day (200 IU/kg/day) with continued clinical improvement and normalization of serum vitamin A levels. Following a two-month period of supplementation, the patient exhibited a complete resolution of ocular surface abnormalities (Figure 1D), accompanied by an improvement in vision to 20/20 in both eyes. The conjunctival surface manifested as smooth, moist, and transparent, devoid of any indications of inflammation. Repeat ERG also showed normalization of rod response (Figure 1E) and follow-up infrared photography demonstrated gradual improvement of the previously abnormal conjunctival patterns (Figure 2B,C). During the treatment course, triangular-shaped areas of persistent conjunctival xerosis were observed, representing classic Bitot’s spots. These spots are characteristic of severe, chronic vitamin A deficiency and typically represent the final areas to resolve during treatment. Based on our clinical observation and the chronic nature of this patient’s dietary restrictions, we hypothesize that the formation of Bitot’s spots [4] is not an acute manifestation but rather may develop through repeated cycles of healing and deterioration in the setting of prolonged inadequate vitamin A intake, ultimately progressing to these characteristic keratinized patches. This case exemplifies the efficacy of infrared photography as a non-invasive diagnostic instrument for identifying early conjunctival keratinization changes in VAD. The technique offers several advantages over conventional examination methods, including enhanced visualization of subtle abnormalities, a non-invasive nature suitable for patients with heightened ocular surface sensitivity [5], objective monitoring of treatment response, and cost-effectiveness using readily available infrared filters adapted to standard ophthalmic cameras. The utilization of infrared imaging functioned as both a diagnostic instrument and a method of objectively monitoring treatment response, thereby unveiling alterations that exhibited a correlation with symptomatic improvement. As a vital component of the human diet, vitamin A plays a pivotal role in maintaining optimal immune function, ensuring optimal visual acuity, and facilitating the differentiation of cells in various physiological processes. Its deficiency has been linked to an elevated risk of infections and is necessary for the production of rhodopsin in retinal rod cells, with deficiency resulting in night blindness. Retinoic acid receptors and retinoid X receptors are critical mediators of vitamin A-regulated cell differentiation. In the absence of vitamin A, there is an increased propensity for keratinization of the skin and ocular surface, which can lead to irreversible corneal clouding and blindness if left untreated [4]. This case demonstrates that severe VAD can occur in developed countries due to selective eating patterns, even when physical growth appears reassuring. Infrared photography is a promising diagnostic modality in ophthalmology that has not been widely adopted, particularly for screening programs in resource-constrained settings. This technique has the potential to serve as a valuable adjunct to conventional examination methods for detecting subtle conjunctival changes and monitoring treatment response in VAD. Future studies comparing infrared photography with anterior segment OCT in larger patient cohorts would be valuable to establish their complementary roles in clinical practice and to develop standardized interpretation criteria. The simplicity and accessibility of this method render it particularly well-suited for widespread clinical adoption, especially in settings where the early recognition and monitoring of nutritional deficiencies are imperative in averting permanent visual loss.

## Data Availability

The data presented in this study are available upon request from the corresponding author.

## References

[1] Manivannan A., Kirkpatrick J.N., Sharp P.F., Forrester J.V. (1994). Clinical investigation of an infrared digital scanning laser ophthalmoscope. Br. J. Ophthalmol..

[2] Dallow R.L. (1974). Color infrared photography of the ocular fundus. Arch. Ophthalmol..

[3] Toslak D., Liu C., Alam M.N., Yao X. (2018). Near-infrared light-guided miniaturized indirect ophthalmoscopy for nonmydriatic wide-field fundus photography. Opt. Lett..

[4] Fukuoka H., Yokoi N., Sotozono C. (2023). Immunohistochemistry in an adult case of Bitot’s spots caused by vitamin A deficiency. Diagnostics.

[5] Courrier E., Lambert V., Renault D., Garcin T., Moine B., Herbepin P., Thuret G., Gain P. (2020). Tolerance to light of patients suffering from infectious keratitis. Cornea.

